# Metagenomics analysis reveals presence of the Merida-like virus in Georgia

**DOI:** 10.3389/fmicb.2023.1258810

**Published:** 2023-10-12

**Authors:** Jennifer M. Potter-Birriel, Adam R. Pollio, Brian D. Knott, Tamar Chunashvili, Christian K. Fung, Matthew A. Conte, Drew D. Reinbold-Wasson, Jun Hang

**Affiliations:** ^1^Walter Reed Army Institute of Research, Silver Spring, MD, United States; ^2^U.S. Army Medical Research Directorate – Georgia (USAMRD-G), Walter Reed Army Institute of Research, Tbilisi, Georgia

**Keywords:** mosquito, *Culex pipiens*, pathogen discovery, vector control, Merida like-virus, NGS

## Abstract

Arbovirus surveillance is fundamental for the discovery of novel viruses and prevention of febrile vector-borne illnesses. Vector-borne pathogens can rapidly expand and adapt in new geographic and environmental conditions. In this study, metagenomic surveillance was conducted to identify novel viruses in the Country of Georgia. A total of 521 mosquitoes were captured near a military training facility and pooled from species *Culex pipiens* (Linnaeus) (87%) and *Aedes albopictus* (Skuse) (13%). We decided to further analyze the *Culex pipiens* mosquitoes, due to the more extensive number of samples collected. Our approach was to utilize an unbiased total RNA-seq for pathogen discovery in order to explore the mosquito virome. The viral reads from this analysis were mostly aligned to Insect-specific viruses from two main families, the *Iflaviridae*; a positive-stranded RNA virus and the *Rhabdoviridae*; a negative- and single-stranded RNA virus. Our pathogen discovery analysis revealed viral reads aligning to the Merida-like virus Turkey (MERDLVT) strain among the *Rhabdoviridae*. To further validate this result, we conducted a BLAST sequence comparison analysis of our samples with the MERDLVT strain. Our positive samples aligned to the MERDLVT strain with 96–100% sequence identity and 99.7–100% sequence coverage. A bootstrapped maximum-likelihood phylogenetic tree was used to evaluate the evolutionary relationships among these positive pooled specimens with the (MERDLVT) strain. The Georgia samples clustered most closely with two strains from Turkey, the Merida-like virus KE-2017a isolate 139-1-21 and the Merida-like virus Turkey isolate P431. Collectively, these results show the presence of the MERDLVT strain in Georgia.

## Introduction

Vector-borne pathogens (VBPs) are one of the main causes of human infections worldwide (Kilpatrick and Randolph, 2012; de Almeida et al., 2021). The geographic boundaries of these vectors continue to expand and adapt around the world’s changing climate (Kilpatrick and Randolph, 2012). For this reason, pathogen discovery becomes increasingly important for arbovirus surveillance and the prevention of VBPs. The most prominent and emerging viruses belong to the *Flaviviridae* family. Flaviviruses are positive-stranded RNA viruses, that are recognized to have the capacity to globally infect and cause a spectrum of several diseases (Pierson and Diamond, 2020; Cuevas-Juarez et al., 2021). This family include several significant human pathogens including Yellow Fever virus (YFV), Zika virus (ZIKV), and Dengue virus (DENV) (Pierson and Diamond, 2020; Cuevas-Juarez et al., 2021). Another major vector-borne virus family, *Rhabdoviridae*, are single-stranded negative RNA viruses, characterized for being ubiquitous, with the ability to infect a wide range of species including plants, vertebrates, and invertebrates (Dietzgen et al., 2017). Two well-known rhabdoviruses include the rabies virus which causes disease in various animals and the infectious hematopoietic necrosis virus which causes disease in salmonid fish. Rhabdoviruses have been shown to exhibit rapid mutation rates and complex genome evolution including gains and losses of genes (Walker et al., 2015). This constant evolution of new variants has made it difficult to the scientific community to develop new vaccines. For this reason, efforts to expand mosquito-borne surveillance in developing countries must continue to be a main priority to keep up with the evolution of emerging viral diseases.

The advances of sequencing metagenomic analysis has led to the discovery of new viruses worldwide, leading to the detection and prevention of emerging viruses. A group of viruses that continues to be detected in mosquitoes are the Insect-specific viruses (ISVs). ISVs can naturally infect and replicate in arthropod hosts. An example of ISVs is the Culex Iflavi-like virus 4 which belongs to the *Iflaviridae* family; a positive-stranded RNA virus (Valles et al., 2017). In recent studies, researchers conducted studies to understand how these ISVs interact with pathogenic arboviruses and how it can be of benefit for vector borne disease control. Continued research will help to develop biotechnology tools that can serve as vector controls to reduce or restrict arboviral diseases (Liu et al., 2011; Bolling et al., 2012; Agboli et al., 2019). In addition, other groups are using ISVs as expression tools against arboviruses for the development of new vaccines (Erasmus et al., 2018). ISVs is gaining interest as potential vectors to restrict pathogenic hosts due to its inability to infect vertebrates (Agboli et al., 2019).

Here, we report the presence of the Merida virus in Senaki, a town within the country of Georgia. The Merida virus was discovered in *Culex pipiens* mosquitoes collected in various sites of a Georgia military facility. Metagenomic analysis was executed using the Chan Zuckerberg ID (CZ ID) cloud-based pipeline; results were further evaluated using phlyogenetics to determine the likely evolutionary origin of our samples. The Merida virus is classified as a rhabdovirus, a single-stranded, negative RNA virus that was originally discovered in the Yucatan Peninsula of Mexico (Charles et al., 2016).

## Materials and methods

### Mosquito collection

Adult host seeking mosquitoes were collected using multiple traps: BG Sentinel 2 (Biogents AG, Regensburg, Germany), CDC light trap (Model 1,012 and 1,212, John W. Hock Company, Gainesville, FL), Stealth Trap (Model 214, John W. Hock Company, Gainesville, FL), and Fay-Prince Trap (Model 812, John W. Company, Gainesville, FL). Collection traps were placed on a tree that was either next to a pond or a pile of tires. Larval dipping collection focused on small water filled containers and larger water pools in order to collect immature mosquitoes. The larval mosquitoes were then allowed to mature in sealed containers then collected as adults. Mosquito collections were conducted between August 2018 and June 2019, in the town of Senaki, located in the Samegrelo-Zemo Svaneti region of Georgia. Mosquitoes were morphologically identified using a stereomicroscope (Leica S4E, Leica microsystems, Germany) and the ECDC MosKey Tool.^1^ Female mosquitoes were sorted into pools of no more than 20 individuals and stored at −80°C. Specimens were shipped frozen from Tbilisi, Georgia to Silver Spring, Maryland, United States. Where they were processed for advanced molecular characterization at the Walter Reed Army Institute of Research, Viral Detection Branch (WRAIR VDB).

### RNA extraction of mosquito pools

Mosquitos were homogenized by bead-beating with the Bio Spec Mini-Bead Beater 16 (Bio Spec Products Inc., Bartlesville, OK, United States). The mosquitos’ homogenates were centrifuged, and the supernatant was treated with DNase I, Benzonase nuclease and RNase A as described in Sanborn et al. (2019). Lysis and RNA extraction was performed using the 5XMag MAX Pathogen RNA/DNA kit (Cat# 4462359) with the Thermo Scientific Kingfisher Flex by following the manufacturer’s user guides.

### RNA amplification, library preparation, and sequencing

Random reverse transcription and PCR amplification (RT-PCR) was conducted on the purified nucleic acid as described by Sanborn et al. (2019). The RT-PCR amplicons were purified and quantified using the Quant-iT^™^ Pico Green^™^ dsDNA Assay (Thermo Fisher Scientific). Library prep was made using the DNA Prep library prep kit (Illumina, San Diego, CA, United States) (Product # 20060059). Libraries were quantitated using Tape Station, D5000 Screen Tape (Agilent Technologies, Inc., Santa Clara, CA, United States), pooled at equal molar concentrations, and sequenced using Illumina MiSeq system and Sequencing Reagent Kit v3 (600-cycle) (Product # MS-102-3003).

### Metagenomics data analyses

Data analysis was performed by uploading MiSeq raw sequence read data to CZID.org (Ramesh et al., 2019; Saha et al., 2019; Kalantar et al., 2020) followed by the metagenomic analysis function within the site. The data was then sorted by reference from the NCBI database. Heat maps were also generated by CZID.org.

We further verified our results by using our in-house *de novo* pipeline previously described (Kilianski et al., 2015).This is a preprocessing step using Cutadapt (Martin, 2011) and Prinseq (Schmieder and Edwards, 2011), followed by a de-novo assembly using RAY Meta (Boisvert et al., 2012). The resulting contigs are then further combined using Cap3 (Huang and Madan, 1999). The final scaffold is then categorized with iterative BLAST against the NCBI nucleotide database (nr/nt).

The Merida-like virus KE-2017a (accession number NC_040532.1) was used as a reference to map sequence reads on Geneious prime. The resulting sequences were then aligned to remove sequencing artifacts followed by a BLAST to show sequence similarity and identity. We then identified the individual ORFs using BLASTp for our annotation.

RASTtk (Brettin et al., 2015) with the “correct frame shift” option was used to annotate the assembled genome sequence. We referenced Merida virus (Taxon ID: 1803034) to build and identify ORFs. We then compared those results using the protein search tool BLASTp (Altschul et al., 1990). Proteins with low similarity were not reported.

### Phylogenetic analyses

We compared 10 whole genome Merida-like virus (Georgia) sequences with 30 publicly available Merida-like viruses sequences, including Hirame rhabdovirus, Infectious hematopoietic necrosis virus, Zahedan rhabdovirus, Long Island tick rhabdovirus, Moussa virus, Arborteum virus, Puerto Almendras virus, Bovine ephemeral fever virus, Kimberley virus, Coastal Plain virus, Tibrogargan virus, Shayang Fly Virus 2 strain, *Drosophila melanogaster* sigmavirus AP30, Wuhan Louse Fly Virus 9 strain, Iriri virus nucleoprotein, European bat 1 lyssavirus, Australian bat lyssavirus, Rabies virus, Bole Tick Virus 2 strain, Culex tritaeniorhynchus rhabdovirus, Culex rhabdovirus strain CRV (Ticino, Switzerland), Merida virus isolate XY14959 nucleoprotein, Merida virus isolate CC_H, Culex rhabdovirus strain CRV (Kern, California), Merida virus isolate MERDV-2020, Merida virus isolate CMS002, Merida virus isolate MERD-Mex07, Merida virus OTU4, Merida-like virus KE-2017a isolate, and Merida-like virus isolate P431. The sequences were aligned using MAFFT version v7.475 using the high-speed setting (Katoh and Standley, 2013). This alignment quality was inspected and confirmed in Geneious, and a phylogenetic tree was constructed using IQ-TREE version 2.0.3 with Model Finder to determine the most appropriate model and the following specific settings: “-ninit 2 -bb 1,000 -nt 4” (Kalyaanamoorthy et al., 2017; Hoang et al., 2018). The resulting tree was edited using FigTree version 1.4.4.

## Results and discussion

### Mosquito collections in the country of Georgia

**Figure 1:**
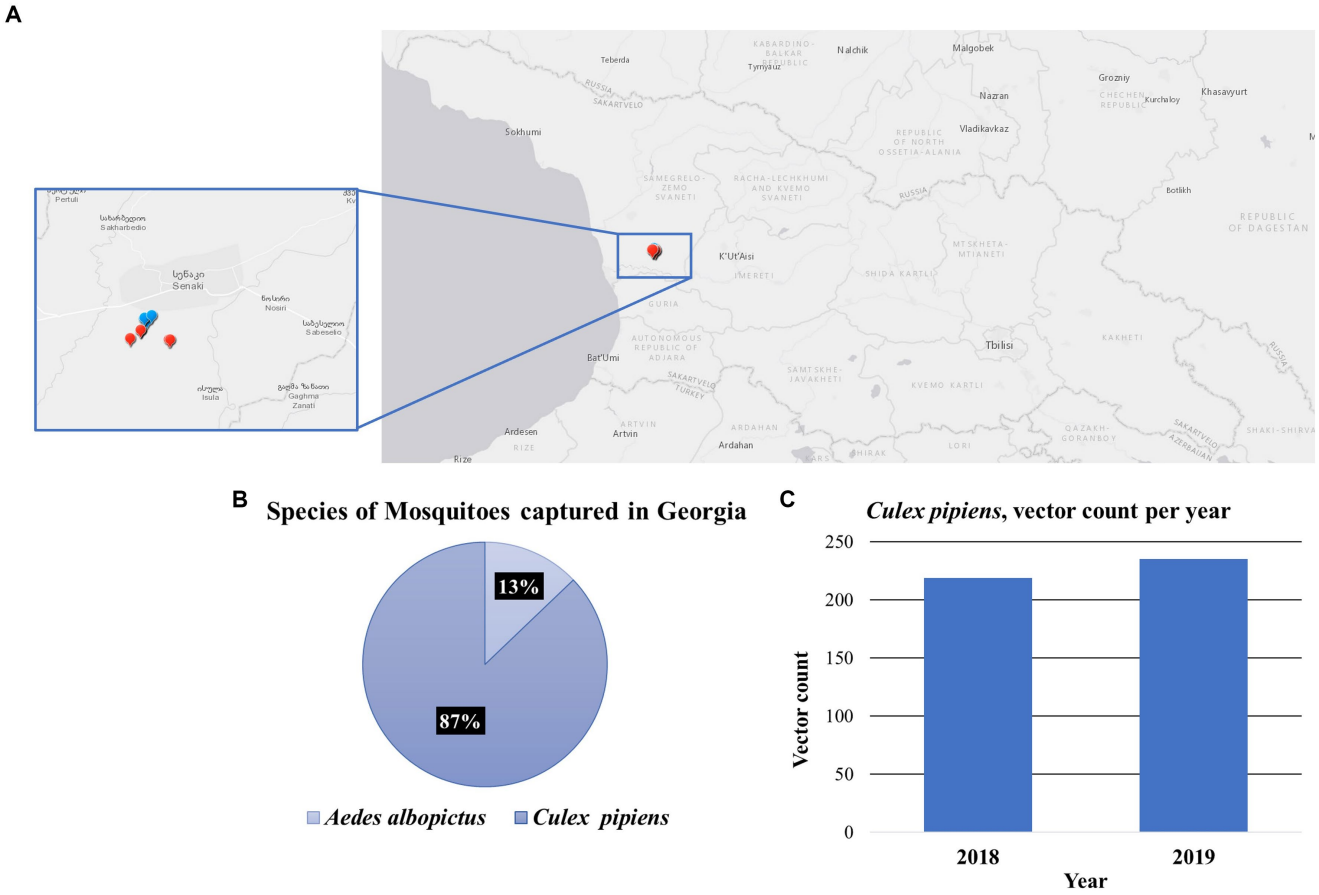
Mosquito collections in the Country of Georgia. **(A)** A map containing the location for each collection site, in the Senaki area. Each dot represents the collection site, 2018 (blue) and 2019 (red). **(B)** Species of mosquitoes captured in Georgia. Chart illustrates the percentage of the two species of mosquitoes captured, the *Culex pipiens* 87% and the *Aedes albopictus* 13%. **(C)** The vector count of *Culex pipiens* mosquitoes captured per year, 2018 (*N* = 219) and 2019 (*N* = 235).

Mosquito sampling was conducted at 10 sites in Senaki, a town in Samegrelo-Zemo Svaneti region located in western Georgia. These collections were made in 2018 and 2019 on a Georgia military training base, the coordinates of each site were recorded using the Global Positioning System (GPS) (Figure 1A; Supplementary Table S1). A total of 521 mosquitoes were captured and distributed into 45 pools from species *Culex pipiens* (87%) and *Aedes albopictus* (13%) (Figure 1B). Due to the more extensive number of samples collected, we decided to further analyze *Culex pipiens* mosquitoes. Figure 1C illustrates the number of *Culex pipiens* mosquitoes collected each year.

### Detection of the Merida-like virus KE-2017a in pooled specimens collected in Senaki

**Figure 2:**
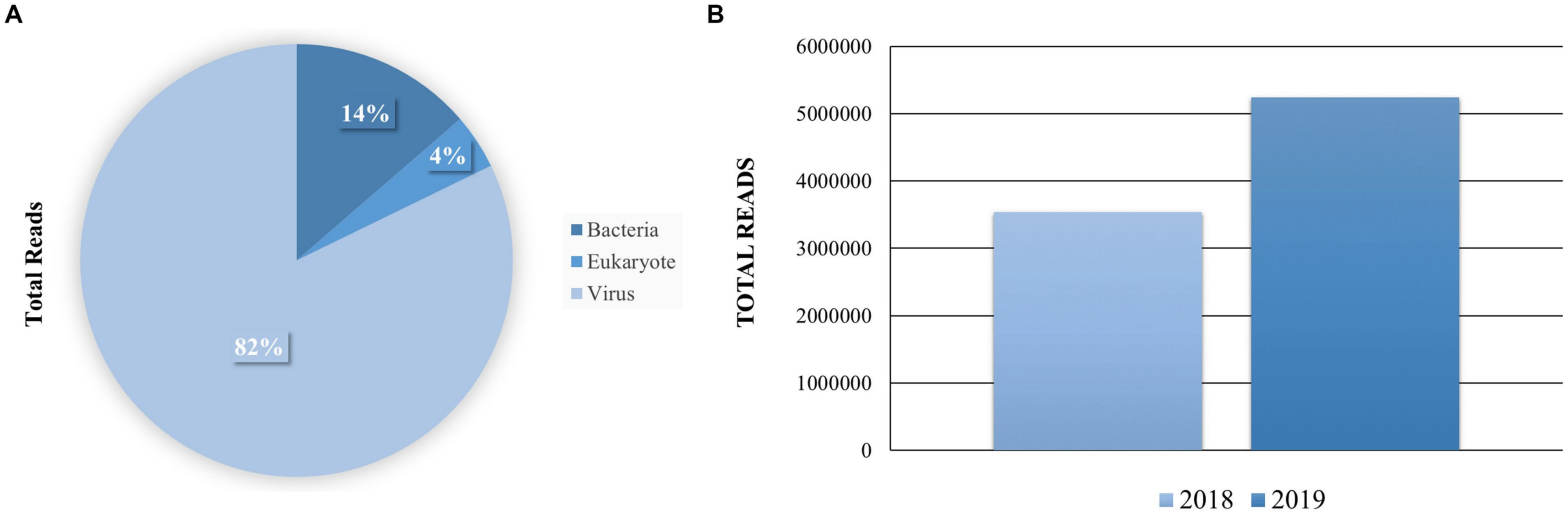
Distribution of the total reads per sample and taxonomy kingdom. **(A)** The total reads per sample were queried to the NCBI database using CZ ID and STAR. The mapped Super kingdom data is shown Bacteria 1,430,993 (14%), Eukaryote 445,670 (4%) and Virus 8,775,711 (82%). **(B)** Comparison of sequencing reads that were aligned to viruses per sample.

To explore the viral diversity of the town of Senaki, we conducted an unbiased total RNA-seq approach to identify viruses in this specific area. This methodology was previously described and published by us (Hang et al., 2012; Sanborn et al., 2019; Pollio et al., 2022). A total of 37 pooled samples were prepared using the Illumina sequencing system. To analyze our Next-generation sequencing (NGS) raw data, we utilized the CZ ID cloud-based pipeline, a computational tool to detect microbial pathogens (Kalantar et al., 2020). With this computational tool, we obtained an average read passing filter per sample of 203,822. Sequences were aligned to the NCBI’s database of nucleotide sequences to calculate rPM (reads per million) and classified into three kingdoms. The total reads per kingdom are the following: Bacteria (1,430,993,14%), Eukaryote (445,670, 4%) and Virus (8,775,711, 82%) (Figure 2A). Most of our sequencing reads were aligned to viruses (82%); thus, we decided to further explore and compare the viral diversity between the years 2018 and 2019 (Figure 2B).

**Figure 3:**
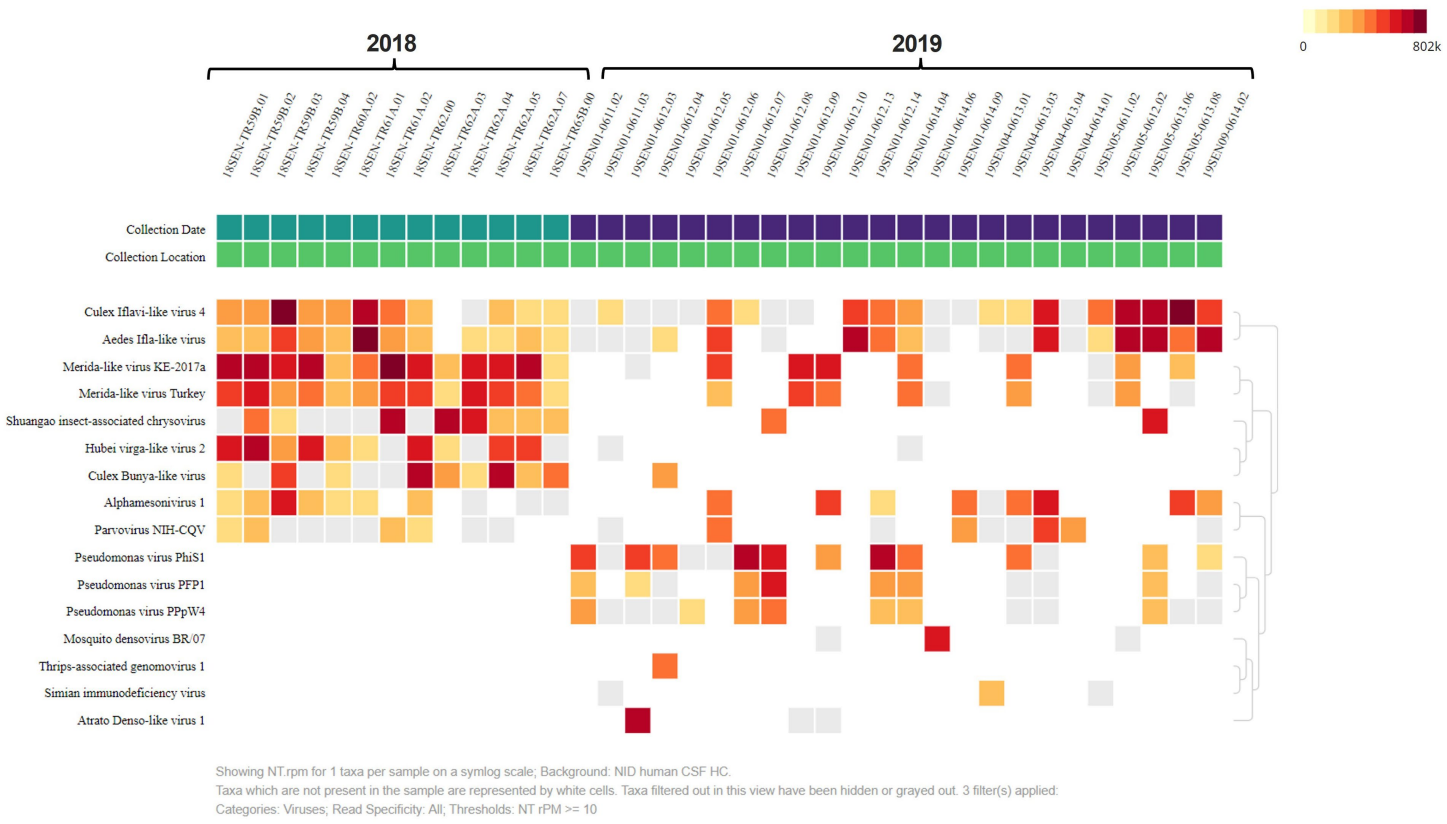
Heat Map analysis shows virus diversity in collected samples. The heat map was set to NT rPM > =10 which only shows viruses with at least 10 reads per million. Samples are divided by collection date. The most abundant viruses aligned to insect-specific viruses including: the Culex Iflavi-like virus 4, Merida-like virus KE-2017a and the Merida-like virus Turkey.

A heat map was built, to analyze the presence and abundance of viral reads in our samples. The heat map was set to NT rPM > =10 which shows viruses with at least 10 reads per million aligned reads to simplify our overall analysis (Figure 3). The most abundant viruses aligned to ISVs including: the Culex Iflavi-like virus 4 (2,167,511.86), and the Merida-like virus KE-2017a (1,304,966.73) (Supplementary Figure S1). These results indicate the abundance of two main families in our pooled samples: the *Iflaviridae*; a positive-stranded RNA virus and the *Rhabdoviridae*; a single-stranded, negative RNA virus. We found the Merida-like virus KE-2017a in most of the collected pooled samples from 2018 (Figure 3). The Merida virus was first detected in the *Culex quinquefasciatus* species in the Yucatan Peninsula of Mexico and classified as a Rhabdovirus (Charles et al., 2016). This virus has been detected in other places around the world (Charles et al., 2016; Ergunay et al., 2017; Sadeghi et al., 2018; da Silva Neves et al., 2021). Airborne surveillance conducted in Thrace and Anatolia, two regions from Turkey recently identified the Merida-like virus Turkey (MERDLVT) (Ergunay et al., 2017). This virus is closely related to the Merida virus detected in Mexico. Due to the proximity of Georgia to Turkey, these results could imply a migration of the Merida virus.

### Evolutionary analysis confirms the presence of the Merida-like virus

**Figure 4:**
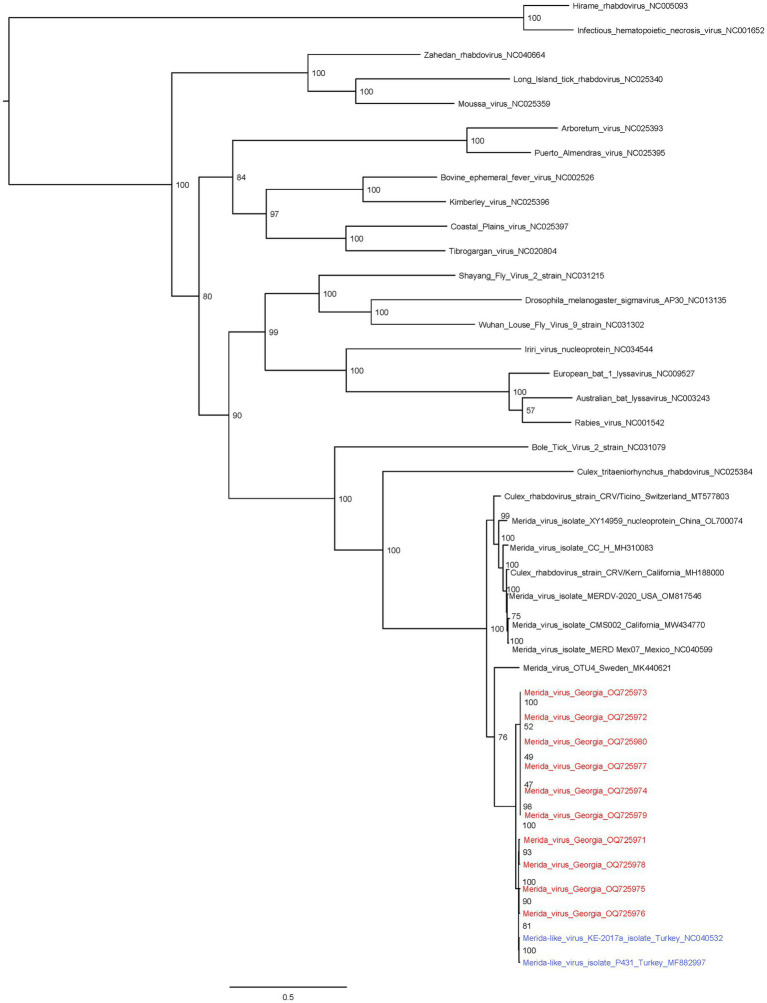
Maximum likelihood tree of rhabdovirus family including Merida virus and Merida-like virus samples. Merida virus genomes from Georgia samples are highlighted in red and closely related MERDLVT samples are highlighted in blue. Node confidence values represent ultrafast bootstrap replicate support.

We used phylogenetic tools to further evaluate the relationship of these positive pooled specimens with the Merida virus. The phylogenetic analysis indicates that our positive samples are closely related to two viruses from mosquitoes collected in Turkey, the Merida-like virus KE-2017a and the MERDLVT (Figure 4). 81% of ultrafast bootstrap replicates supported the common ancestor node that places the two samples from Turkey with two samples from Georgia (Merida_virus_Georgia_OQ725976 and Merida_virus_Georgia_OQ725975). To further confirm the previous analysis, we used the BLAST sequencing alignment tool to evaluate and compare the sequence identity between the Georgia samples and the MERLDVT strain. Our positive samples aligned to the MERDLVT strain with 96%–100% sequence identity with 99.7%–100% aligned genome sequence coverage.

Our aim was to explore unique viral communities to expand our knowledge in mosquito virome populations. To explore the viral component of the town of Senaki we used an unbiased metagenomic approach. Pooled specimens of *Culex pipiens* mosquitoes were studied to reveal mosquito virome. In both years, most viral reads belonged to the Culex Iflavi-like virus 4. In addition, we found most samples containing the Merida-like virus KE-2017a in pooled specimens from 2018. A phylogenetic analysis revealed that these positive pooled samples shared a common ancestor with the Merida-like virus KE-2017a isolate 139-1-21 and the Merida-like virus Turkey isolate P431. The known Merida viruses shown in this phylogenetic analysis cluster geographically.

The Merida virus was initially detected in *Culex quinquefasciatus* mosquitoes and assigned to the genus Merhavirus within the *Rhabdoviridae* family (Charles et al., 2016). Since then, the virus has been detected in metagenomic analyses conducted in other mosquito species and around the world (Charles et al., 2016; Ergunay et al., 2017). However, the pathology of the Merida virus has not been further explored.

Controlling vector populations has become widely implemented approach to prevent the endemic spread of VBPs. With the continuous expansion of vector habitats, it is critical to monitor vector populations for potential emerging viral diseases to better prepare for new threats. Our work has added contributed NGS data and curated genome assemblies of novel Merida-like viruses that can be further used to better understand viral communities within the mosquito population. These data have the potential in combination with additional surveys to identify new and emerging diseases.

## Data availability statement

The datasets presented in this study can be found in online repositories. The names of the repository/repositories and accession number(s) can be found at: https://www.ncbi.nlm.nih.gov/genbank/, OQ725971; https://www.ncbi.nlm.nih.gov/genbank/, OQ725972; https://www.ncbi.nlm.nih.gov/genbank/, OQ725973; https://www.ncbi.nlm.nih.gov/genbank/, OQ725974; https://www.ncbi.nlm.nih.gov/genbank/, OQ725975; https://www.ncbi.nlm.nih.gov/genbank/, OQ725976; https://www.ncbi.nlm.nih.gov/genbank/, OQ725977; https://www.ncbi.nlm.nih.gov/genbank/, OQ725978; https://www.ncbi.nlm.nih.gov/genbank/, OQ725979; https://www.ncbi.nlm.nih.gov/genbank/, OQ725980.

## Author contributions

JP-B: Formal analysis, Methodology, Writing – original draft, Writing – review & editing, Conceptualization, Investigation, Visualization. AP: Formal analysis, Methodology, Writing – original draft, Writing – review & editing, Conceptualization, Investigation. BK: Methodology, Project administration, Writing – review & editing, Investigation, Formal analysis. TC: Methodology, Writing – review & editing, Investigation. CF: Formal analysis, Methodology, Software, Visualization, Writing – review & editing, Validation. MC: Formal analysis, Methodology, Writing – review & editing, Software, Visualization, Validation. DR-W: Formal analysis, Funding acquisition, Methodology, Project administration, Supervision, Writing – review & editing, Investigation, Resources. JH: Formal analysis, Funding acquisition, Methodology, Project administration, Supervision, Writing – review & editing, Investigation, Resources, Validation, Visualization.

## Footnotes

^1^ https://www.medilabsecure.com/moskeytool.html

## Author disclaimer

Material has been reviewed by the authors’ respective institutions. There is no objection to its presentation and/or publication. The views expressed here are those of the authors and do not reflect the official policy of the Department of the Army, Department of Defense or US Government. This is the work of US government employees and may not be copyrighted (17 USC 105).
