# Supplementary material for: Metagenomics analysis reveals presence of the Merida-like virus in Georgia
Source: Front Microbiol. 2023 Oct 12;14:1258810. doi: 10.3389/fmicb.2023.1258810 (PMC10602647; doi:10.3389/fmicb.2023.1258810)

**Supplementary Material**

**Metagenomics analysis reveals presence of the Merida-like virus in Georgia**

Jennifer M. Potter-Birriel^1^ ^†^, Adam R. Pollio^1†^, Brian D. Knott^2^, Tamar Chunashvili^2^, Christian K. Fung^1^, Matthew A. Conte^1^, Drew D. Reinbold-Wasson^2^, Jun Hang^1^

^†^ These authors contributed equally to this work and share first authorship.

**Correspondence:**

Corresponding Author: Jun Hang, PhD

[jun.hang.civ@health.mil](mailto:jun.hang.civ@health.mil)

1. **Supplementary Table and Figure**
   1. **Supplementary Table**

**Supplementary Table 1.** Metadata for each sample collected. Description of the collection method, year of collection, vector count, for each pooled sample collected in Senaki, Samegrelo-Zemo Svaneti in August 2018 and June 2019.

| Sample name | Vector ID | Collecting Method / Trap Type | Collection  Date | Vector Count |
| --- | --- | --- | --- | --- |
| 18Steg 26-25.01 | *Aedes albopictus* | Larva Dipping | 16/6/2018 | 13 |
| 18SEN-TR56A.00 | *Aedes albopictus* | BG Sentinel 2 | 19/8/2018 | 1 |
| 18SEN-TR59.00 | *Aedes albopictus* | BG Sentinel 2 | 18/8/2018 | 16 |
| 18SEN-TR59.01 | *Aedes albopictus* | BG Sentinel 2 | 18/8/2018 | 15 |
| 18SEN-TR59B.01 | *Culex pipiens* | BG Sentinel 2 | 20/8/2018 | 8 |
| 18SEN-TR59B.02 | *Culex pipiens* | BG Sentinel 2 | 20/8/2018 | 20 |
| 18SEN-TR59B.03 | *Culex pipiens* | BG Sentinel 2 | 20/8/2018 | 20 |
| 18SEN-TR59B.04 | *Culex pipiens* | BG Sentinel 2 | 20/8/2018 | 17 |
| 18SEN-TR60A.02 | *Culex pipiens* | CDC Light Trap | 19/8/2018 | 19 |
| 18SEN-TR61A.01 | *Culex pipiens* | CDC Light Trap | 19/8/2018 | 21 |
| 18SEN-TR61A.02 | *Culex pipiens* | CDC Light Trap | 19/8/2018 | 20 |
| 18SEN-TR62.00 | *Culex pipiens* | BG Sentinel 2 | 18/8/2018 | 19 |
| 18SEN-TR62A.03 | *Culex pipiens* | BG Sentinel 2 | 19/8/2018 | 20 |
| 18SEN-TR62A.04 | *Culex pipiens* | BG Sentinel 2 | 19/8/2018 | 20 |
| 18SEN-TR62A.05 | *Culex pipiens* | BG Sentinel 2 | 19/8/2018 | 18 |
| 18SEN-TR62A.07 | *Culex pipiens* | BG Sentinel 2 | 19/8/2018 | 10 |
| 18SEN-TR65B.00 | *Culex pipiens* | CDC Light Trap | 20/8/2018 | 7 |
| 19SEN09-0614.02 | *Culex pipiens* | Fay-Prince Trap - CDC | 14/6/2019 | 10 |
| 19SEN04-0613.01 | *Culex pipiens* | Fay-Prince Trap - CDC | 13/6/2019 | 10 |
| 19SEN04-0613.03 | *Culex pipiens* | Fay-Prince Trap - CDC | 13/6/2019 | 7 |
| 19SEN04-0613.04 | *Culex pipiens* | Fay-Prince Trap - CDC | 13/6/2019 | 7 |
| 19SEN04-0614.01 | *Culex pipiens* | Fay-Prince Trap - CDC | 14/6/2019 | 10 |
| 19Steg0611-1801.01 | *Aedes albopictus* | Larva Dipping | 11/6/2019 | 10 |
| 19Steg0611-1801.02 | *Aedes albopictus* | Larva Dipping | 11/6/2019 | 10 |
| 19SEN01-0611.02 | *Culex pipiens* | Stealth Trap - JW Hock | 11/6/2019 | 10 |
| 19SEN01-0611.03 | *Culex pipiens* | Stealth Trap - JW Hock | 11/6/2019 | 13 |
| 19SEN01-0612.03 | *Culex pipiens* | Stealth Trap - JW Hock | 12/6/2019 | 10 |
| 19SEN01-0612.04 | *Culex pipiens* | Stealth Trap - JW Hock | 12/6/2019 | 10 |
| 19SEN01-0612.05 | *Culex pipiens* | Stealth Trap - JW Hock | 12/6/2019 | 10 |
| 19SEN01-0612.06 | *Culex pipiens* | Stealth Trap - JW Hock | 12/6/2019 | 10 |
| 19SEN01-0612.07 | *Culex pipiens* | Stealth Trap - JW Hock | 12/6/2019 | 10 |
| 19SEN01-0612.08 | *Culex pipiens* | Stealth Trap - JW Hock | 12/6/2019 | 10 |
| 19SEN01-0612.09 | *Culex pipiens* | Stealth Trap - JW Hock | 12/6/2019 | 10 |
| 19SEN01-0612.10 | *Culex pipiens* | Stealth Trap - JW Hock | 12/6/2019 | 10 |
| 19SEN01-0612.13 | *Culex pipiens* | Stealth Trap - JW Hock | 12/6/2019 | 10 |
| 19SEN01-0612.14 | *Culex pipiens* | Stealth Trap - JW Hock | 12/6/2019 | 10 |
| 19SEN01-0614.01 | *Aedes albopictus* | Stealth Trap - JW Hock | 14/6/2019 | 1 |
| 19SEN01-0614.04 | *Culex pipiens* | Stealth Trap - JW Hock | 14/6/2019 | 10 |
| 19SEN01-0614.06 | *Culex pipiens* | Stealth Trap - JW Hock | 14/6/2019 | 10 |
| 19SEN01-0614.09 | *Culex pipiens* | Stealth Trap - JW Hock | 14/6/2019 | 10 |
| 19SEN05-0611.02 | *Culex pipiens* | BG Sentinel 2 | 14/6/2019 | 8 |
| 19SEN05-0612.02 | *Culex pipiens* | BG Sentinel 2 | 14/6/2019 | 10 |
| 19SEN05-0613.06 | *Culex pipiens* | BG Sentinel 2 | 13/6/2019 | 10 |
| 19SEN05-0613.08 | *Culex pipiens* | BG Sentinel 2 | 13/6/2019 | 10 |
| 19SEN06-0613.01 | *Aedes albopictus* | Stealth Trap - JW Hock | 13/6/2019 | 1 |

- 1. **Supplementary Figure**

**Supplementary Figure 1.** Insect-specific viruses detected in *Culex pipiens* mosquitoes collected in Georgia. Sequences were aligned for each virus using the NCBI’s database. The Culex Iflavi-like virus 4 and the Merida-like virus KE-2017a are the most abundant viruses detected.


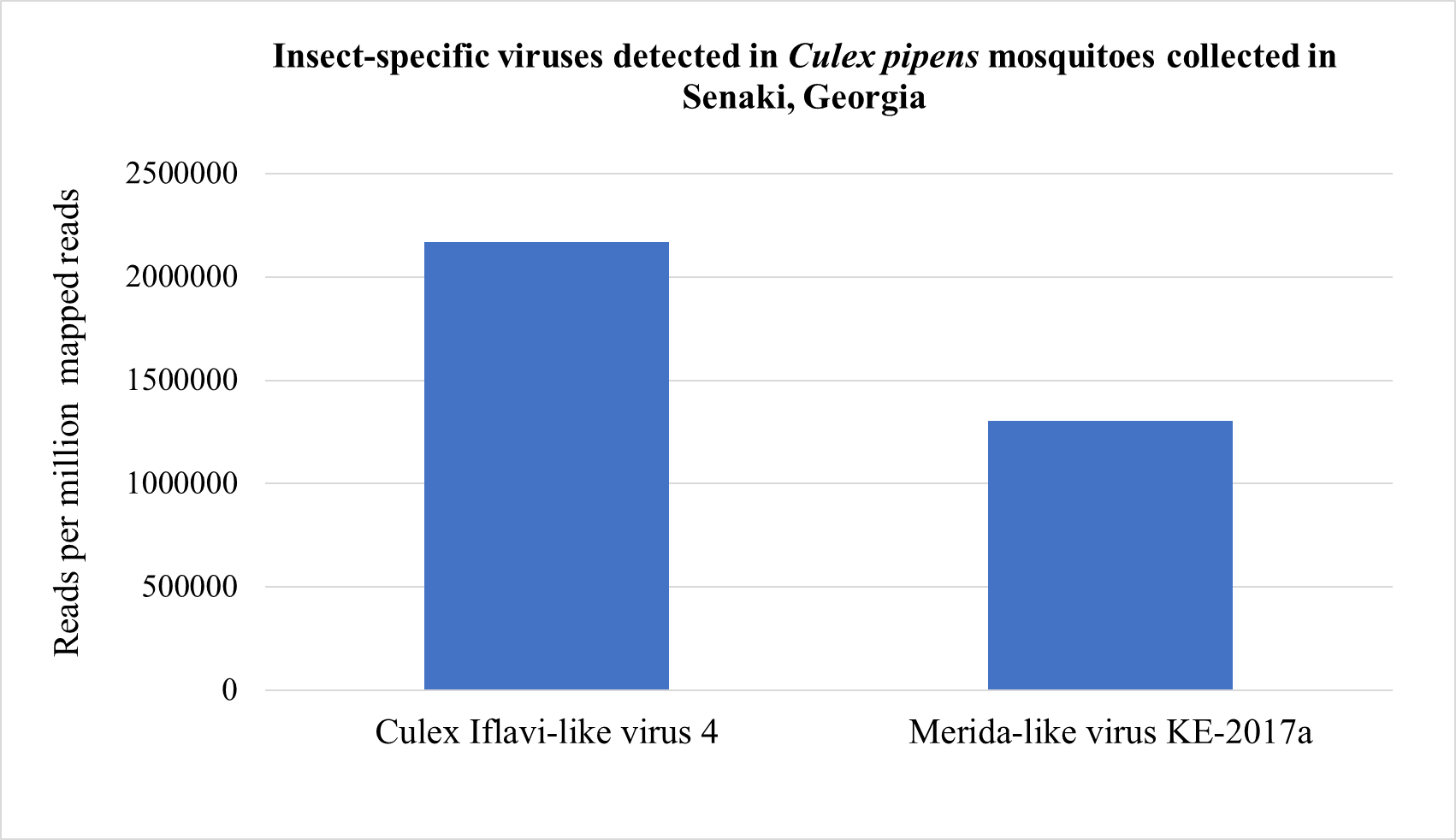

Supplement: Supplementary file 1 [file Data_Sheet_1.docx]
